# CRISPR-Driven Genome Engineering for Chorismate- and Anthranilate-Accumulating *Corynebacterium* Cell Factories

**DOI:** 10.4014/jmb.2305.05031

**Published:** 2023-07-17

**Authors:** Hye-Jin Kim, Si-Sun Choi, Eung-Soo Kim

**Affiliations:** Department of Biological Sciences and Bioengineering, Inha University, Incheon 22212, Republic of Korea

**Keywords:** *Corynebacterium*, chorismate, anthranilate, genome editing, CRISPR

## Abstract

In this study, we aimed to enhance the accumulation of chorismate (CHR) and anthranilate (ANT), key intermediates in the shikimate pathway, by modifying a shikimate over-producing recombinant strain of *Corynebacterium glutamicum* [19]. To achieve this, we utilized a CRISPR-driven genome engineering approach to compensate for the deletion of shikimate kinase (AroK) as well as ANT synthases (TrpEG) and ANT phosphoribosyltransferase (TrpD). In addition, we inhibited the CHR metabolic pathway to induce CHR accumulation. Further, to optimize the shikimate pathway, we overexpressed feedback inhibition-resistant *Escherichia coli* AroG and AroH genes, as well as *C. glutamicum* AroF and AroB genes. We also overexpressed QsuC and substituted shikimate dehydrogenase (AroE). In parallel, we optimized the carbon metabolism pathway by deleting the *gntR* family transcriptional regulator (IolR) and overexpressing polyphosphate/ATP-dependent glucokinase (PpgK) and glucose kinase (Glk). Moreover, acetate kinase (Ack) and phosphotransacetylase (Pta) were eliminated. Through our CRISPR-driven genome re-design approach, we successfully generated *C. glutamicum* cell factories capable of producing up to 0.48 g/l and 0.9 g/l of CHR and ANT in 1.3 ml miniature culture systems, respectively. These findings highlight the efficacy of our rational cell factory design strategy in *C. glutamicum*, which provides a robust platform technology for developing high-producing strains that synthesize valuable aromatic compounds, particularly those derived from the shikimate pathway metabolites.

## Introduction

The shikimate pathway is an essential metabolic route involved in the biosynthesis of critical metabolites containing aromatic moieties in plants, animals, and microorganisms [1, 2]. This pathway also plays a role in the synthesis of structural blocks for compounds such as vitamins, cofactors, and quinones that function as electron carriers [3, 4]. The 3-deoxy-D-arabino-heptulosonate-7-phosphate (DAHP) synthesized through the polymerization reaction of phosphoenolpyruvate (PEP) and erythrose-4-phosphate (E4P) generated from various carbon sources via the glycolysis pathway and pentose phosphate pathway is sequentially converted to chorismate (CHR) through a series of enzyme reactions. CHR is then utilized as a precursor for the synthesis of aromatic amino acids such as anthranilate (ANT) and prephenate, as well as a key intermediate for the synthesis of para-aminobenzoic acid, a precursor for folate synthesis [1, 2, 5, 6]. CHR is primarily utilized as a precursor for salicylic acid, which is required for the synthesis of aspirin and muconic acid [1]. ANT can be used as a food ingredient, in the production of dyes and perfumes, as a crop protection compound, in the synthesis of pharmaceutical compounds, as a platform chemical for plastic production, and as a compound for inhibiting biofilm formation in bacteria [9-11].

Efforts are being made to replace the chemical synthesis of aromatic compounds, including CHR and ANT, as the high quality standards required for their utilization in the pharmaceutical or food industries make the complex and toxic byproduct-producing chemical synthesis methods less desirable [13]. Through the optimization of the shikimate pathway, strains can be utilized for the high production of aromatic amino acids and high-demand aromatic chemicals [14]. High production of tyrosol has also been attempted in *Saccharomyces cerevisiae*, but high production of CHR itself has not yet been achieved [15]. As for research on high production of ANT, studies have reported the production of up to 14 g/l and 26.4 g/l through fed-batch fermentation of a recombinant *E. coli* strain and a *C. glutamicum* strain, in addition to 3.8 mM and 1.5 g/l (11.23 mM) of ANT production from *Pseudomonas putida* strains, respectively [7, 16].

*Corynebacterium glutamicum* has emerged as a versatile production strain in various industries, including food, biofuels, biopolymer production, and the synthesis of high-value compounds like resveratrol [17, 18]. Notably, genome-redesigned *C. glutamicum* strains have demonstrated impressive production capabilities, yielding approximately 38 g/l of muconate and shikimate in a 7-L fed-batch fermentation, highlighting their potential as an ideal bacterial host for synthetic cell factory design [19, 20]. In this study, we aimed to generate rationally designed cell factories for accumulation of CHR and ANT, crucial intermediates in the shikimate pathway, utilizing CRISPR-driven *Corynebacterium* genome engineering.

## Materials and Methods

### Bacterial Strains and Culture Conditions

Table 1 lists all bacterial strains used in this study. *E. coli* DH5α was utilized as the cloning host and grown in Luria-Bertani (LB) medium at 30 or 37°C with the appropriate antibiotics. *C. glutamicum* stains were generally cultured in brain heart infusion (BHI) medium containing 91 g/l sorbitol at 30°C. Agar was added at 15 g/l for plates. All recombinant strains were transformed by electroporation. All strains were kept as glycerol stocks prepared in LB or BHIS broth containing 20% glycerol at -80°C. For CHR and ANT production, a single colony of *C. glutamicum* strains was inoculated in 1.3 ml LB medium containing 10 g/l glucose at 30°C for 15 h, while culture broth was inoculated with 1% (v/v) in the same medium at 30°C for 6 h. A secondary culture broth was inoculated in 1.3 ml production medium at 30°C for 4 days. The production media were described elsewhere [20]. The miniature cultivation was performed using a humidity chamber set at 80% humidity and 200 rpm.

### Construction of Plasmid and Strains

The constructed plasmids are listed in Table 1, and all primer pairs used in this study are displayed in Table S1. For targeted gene editing, the all-in-one CRISPR/Cpf1 plasmid pJYS3 was utilized. N_24_ sequences followed by the PAM were designed by web tool, CHOPCHOP (https://chopchop.cbu.uib.no). The homologous DNA fragments to the upstream and downstream regions of the target gene were amplified with primer sets. The fragment including N_24_ sequence and guide RNA scaffold were also amplified. Then, these fragments were cloned into SmiI/XbaI-digested pJYS3 based on the In-Fusion Cloning method (TaKaRa, Japan). Genome editing and plasmid curing were performed as described previously [61]. Transformants were verified by colony PCR and DNA sequencing. For amplification of target-specific fragments and colony PCR, TransStart FastPfu Fly DNA polymerase (Transgen Biotech., China) and SapphireAmp Fast PCR Master Mix (TaKaRa, Japan) were used, respectively.

### CHR and ANT Analyses

Cultured broth was centrifuged at 15,000 ×*g* for 10 min. The supernatant was filtered using a Nylaflo nylon membrane filter and stored at -20°C. The concentrations of CHR and ANT were determined by high-performance liquid chromatography (HPLC) using a Zorbax SB-Aq column (4.6 x 250 mm, Agilent, USA). The mobile phase was 0.1% trifluoroacetic acid (TFA) in 40% MeOH and the flow rate was 0.5 ml/min. CHR and ANT were detected at 273 nm and 330 nm, respectively.

## Results and Discussion

### Development of the CHR- and ANT-Accumulating *Corynebacterium* Strains

**Fig. 1:**
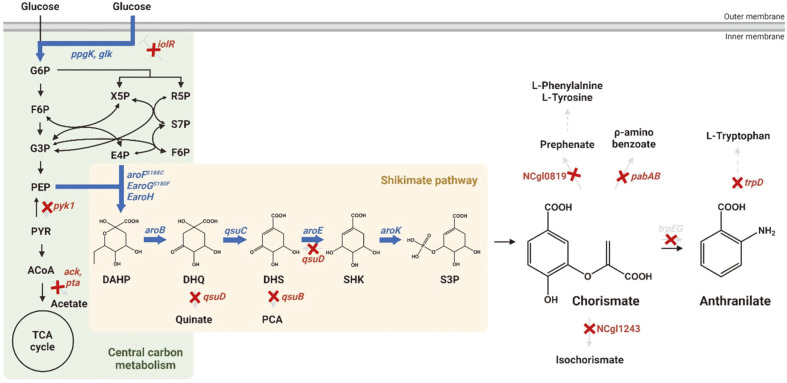
Scheme of pathway engineering for CHR and ANT production in *Corynebacterium glutamicum*. Red crosses indicate disrupted genes and bold blue arrows indicate enhanced steps through target gene overexpression. G6P; glucose-6-phosphate, F6P; fructose-6-phosphate, G3P; glyceraldehyde-3-phosphate, PEP; phosphoenolpyruvate, PYR; pyruvate, ACoA; acetyl-CoA, X5P; xylulose-5-phosphate, R5P; ribose-5-phosphate, S7P; sedoheptulose-7-phosphate, E4P; erythrose-4-phosphate, F6P; fructose-6-phosphate, DAHP; 3-deoxy-D-arabisoheptulosanate-7-phosphate, DHQ; 3- dehydroquinate, DHS; 3-dehydroshikimate, SHK; shikimate, S3P; shikimate-3-phosphate.

**Fig. 2:**
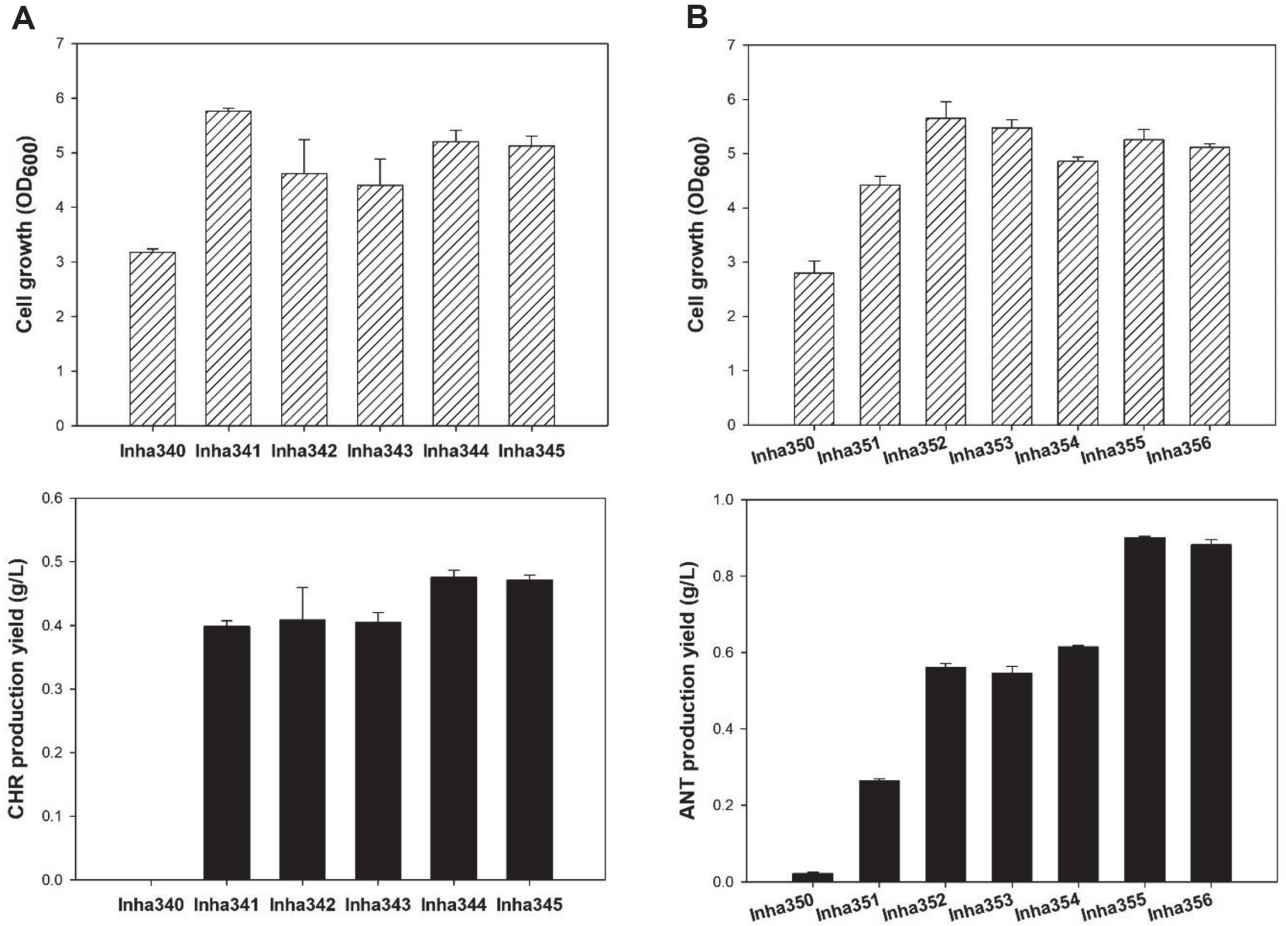
Comparison of cell growth and metabooite production yields in recombinant *C. glutamicum* strains. (**A**) Cell growth as measured by OD_600_ (top), quantitative analysis of CHR titer in CHR-producing *C. glutamicum* strains (Inha340, Inha341, Inha342, Inha343, Inha344 and Inha345). (**B**) Cell growth as measured by OD_600_ (top), quantitative analysis of ANT titer in ANT-producing *C. glutamicum* strains (Inha350, Inha351, Inha352, Inha353, Inha354, Inha355 and Inha356). All assays were performed in triplicate.

In this study, we aimed to develop CHR- and ANT-accumulating strains based on a shikimate-overproducing strain (Inha310), [19] by optimizing the shikimate biosynthesis pathway and carbon metabolism (Fig. 1). Inha310 lacks AroK (NCgl1560), a shikimate kinase involved in the conversion of shikimate to shikimate-3-phosphate, and thus, we first compensated for this by restoring the closed shikimate biosynthesis pathway [19]. For the genome engineering of *C. glutamicum*, a pJYS3 CRISPR vector based on the Cpf1 nuclease system utilizing a T-rich protospacer-adjacent motif (PAM) was used [21]. The constructed strains in this study are listed in Table 1 and Fig. S1. The primers used for strain construction are listed in Table S1. The aroK gene was compensated for under the control of a self-regulating promoter, and the resulting strain was used as a base strain for the production of CHR and ANT (Fig. S2). It is known that CHR in *C. glutamicum* is converted to ANT by ANT synthase I, TrpE (NCgl2927), and ANT synthase II, TrpG (NCgl2928). We constructed a strain that accumulates CHR by deleting these two genes (Figs. S3A and S3C). The resulting strain named Inha340 failed to accumulate CHR in miniature cultivation (Fig. 2A). ANT is metabolized in subsequent steps by ANT phosphoribosyltransferase TrpD (NCgl2929), so we constructed a strain capable of accumulating ANT by removing TrpD in Inha304, a shikimate-producing strain compensating for AroK, and naming it Inha350 (Figs. S3B and S3C). The ANT production yield in Inha350, where only TrpD was removed, was found to be 21.4 mg/l, and the low yield of ANT production was expected due to insufficient accumulation of CHR, which may be metabolized by other CHR metabolic pathways (Fig. 2B).

It is suspected that NCgl0819 is involved in the conversion of CHR to prephenate, PabAB (NCgl0955) in the synthesis of para-aminobenzoate, and EntC (NCgl1243) in the conversion of CHR to isochorismate. To induce the overaccumulation of CHR and ANT, we constructed Inha351 by deleting the genes encoding these three enzymes based on the ANT-producing strain Inha350 (Fig. S4). Through miniature cultivation, we confirmed that approximately 0.26 g/l of ANT was produced from Inha351, and the production yield of ANT was increased 12.3 times compared to Inha350, in which only TrpD was deleted by blocking the CHR metabolic pathways (Fig. 2B). In addition, we constructed a CHR-producing strain, Inha341, by removing TrpEG based on Inha351. We also confirmed that approximately 0.4 g/l of CHR was produced from this strain (Fig. 2A). Moreover, we observed that the cell growth of Inha341 and Inha351, in which both CHR and ANT metabolic pathways were blocked, increased by at least 150% compared to Inha340 and Inha350 strains in which the aromatic amino acid biosynthesis pathways were not blocked (Fig. 2). This can be interpreted as an indication that there may be alternative pathways in *C. glutamicum* that can replace aromatic amino acid biosynthesis through the shikimate pathway.

### Optimizing the Shikimate Pathway for Enhanced CHR and ANT Production

Inha310, a shikimate-overproducing *C. glutamicum* strain, was previously constructed by cloning five shikimate pathway enzymes (AroF, AroG, AroB, QsuC, AroE) into a single plasmid and introducing it into the strain [19]. We redesigned the genome to enable the constitutive expression of shikimate biosynthetic enzymes under the *sod* or *tuf* promoter (the 225 bp upstream of *sod* gene or 248 bp upstream of *tuf* gene, respectively), aiming to enhance the shikimate pathway in a plasmid-free form. In *E. coli*, it is suspected that AroF, AroG, and AroH are involved in the condensation reaction of key precursors, phosphoenolpyruvate (PEP) and erythrose-4-phosphate (E4P), in the shikimate pathway. However, in *C. glutamicum*, AroF and AroG are known to be involved in this reaction. Previous studies have reported that the productivity of L-phenylalanine can be maximized by additionally expressing the *aroH* gene from *E. coli* in *C. glutamicum* [22]. Therefore, to enhance the shikimate pathway, *E. coli* feedback inhibition-resistant AroG (E*aroG*) and AroH (E*aroH*) were substituted for AroF in the *C. glutamicum* genome under the control of the *tuf* promoter (Fig. S5A). The strain, based on high ANT-producing strain Inha351, was named Inha352, and in it, the AroF gene from *C. glutamicum* was replaced with *E. coli* feedback inhibition-resistant AroG (E*aroG*) and AroH (E*aroH*),. In Inha352, the production of 0.56 g/l ANT was confirmed (Fig. 2B).

Feedback inhibition-resistant AroF and AroB were combined and substituted with AroG in the *C. glutamicum* genome, allowing them to be expressed under the *tuf* promoter. (Fig. S5B). Based on Inha352, the strain derived from ANT-producing strain Inha351, *C. glutamicum* feedback inhibition-resistant AroF and AroB were replaced with *C. glutamicum*-derived AroG under the control of the *tuf* promoter (Fig. S5B). The resulting strain, named Inha353, was found to produce 0.55 g/l of ANT (Fig. 2B). In addition, efficient expression of shikimate dehydrogenase, which is involved in the conversion from DHS to shikimate, is essential for strengthening the shikimate biosynthesis pathway. Previous studies have shown that expressing *E. coli*-derived AroE (E*aroE*) in *C. glutamicum* is more efficient for the conversion of DHS to shikimate than overexpressing the native shikimate dehydrogenase (AroE) in *C. glutamicum* [19]. We replaced the *C. glutamicum* native AroE with *E. coli*-derived AroE (E*aroE*) and QsuC under the *sod* promoter to enable their expression in *C. glutamicum* (Fig. S5C) for efficient conversion of DHS to shikimate. Inha354 strain was named by replacing *C. glutamicum*-derived AroE with *E. coli*-derived AroE (E*aroE*) and QsuC under the *sod* promoter based on Inha353. We found that this strain produced 0.61 g/l of ANT (Fig. 2B). CHR-producing strains (Inha342 and Inha343) were constructed by further removing TrpEG based on the three ANT-producing strains (Inha353 and Inha354), and it was confirmed that 0.41 g/l and 0.40 g/l of CHR were produced in these strains (Fig. 2A).

### Engineering the Central Carbon Metabolic Pathway for Enhanced CHR and ANT Accumulation

For high production of the major precursors, PEP and E4P in the shikimate biosynthesis pathway, optimization of carbon metabolism pathways is necessary. In *C. glutamicum*, myo-inositol permease (IolT) involved in glucose transfer along with the phosphotransferase system (PTS) is inhibited by the *gntR* family transcriptional regulator IolR, so deletion of the *iolR* gene can improve glucose uptake into the cell. Furthermore, to improve carbon source uptake into the cell, polyphosphate/ATP-dependent glucokinase (PpgK) or glucose kinase (Glk) involved in the metabolism of glucose transported into the cell by IolT were overexpressed [23]. To prevent unnecessary accumulation of acetate during the shikimate production process, we removed acetate kinase (Ack) and phosphotransacetylase (Pta) involved in acetate accumulation. A strain with *ppgK* and *glk* genes substituted for *iolR* gene, named Inha344, was constructed based on Inha343, a CHR-producing strain. Inha345, a strain with Ack and Pta deleted, was further constructed based on Inha344. Through miniature cultivation, it was confirmed that Inha344 and Inha345 strains produced 0.48 g/l and 0.47 g/l of CHR, respectively (Figs. 2A and S6). Based on the ANT-producing strain Inha354, we constructed strain Inha355 by substituting the *iolR* with *ppgK* and *glk* genes, and further deleted Ack and Pta genes to generate Inha356. Through miniature cultivation, we confirmed that Inha355 and Inha356 strains produced 0.90 g/l and 0.88 g/l of ANT, respectively (Figs. 2B and S6), based on Inha354 strain as a starting point for the substitution of the *ppgK* and *glk* genes with the *iolR* gene and the subsequent deletion of the Ack and Pta genes.

In microbial-based ANT production reported so far, a maximum of 14 g/l has been achieved with recombinant *E. coli*, while a maximum of 26.40 g/l has been reported for single-phase fermentation of *C. glutamicum*. Particularly, the recombinant *C. glutamicum* strain was constructed by replacing the promoters of shikimate kinase AroK and 3-dehydroquinate synthase AroB with a strong *sod* promoter and overexpressing G6P isomerase Pgi and G6P 1-dehydrogenase Zwf, which are involved in carbon metabolism, thereby optimizing the shikimate and carbon metabolic pathways. Based on this, it can be inferred that the overexpression of enzymes involved in the shikimate pathway and carbon metabolism is essential for anthranilate production. It is expected that additional productivity increase could be achieved if specific culture optimization is carried out for the CHR and ANT production strains developed in this study. Furthermore, the CHR-overproducing strain constructed in this study can serve as a foundational strain for the high production of non-aromatic compounds, such as cyclohexadiene-transdiols, in addition to salicylate. Similarly, the ANT- overproducing strain can be utilized as a foundational strain for the high production of useful aromatic compounds, such as aromatic amino acids or methyl ANT.

In summary, the findings presented in this study underscore the immense potential of *C. glutamicum* as a highly efficient cell factory for the accumulation of valuable aromatic compounds, specifically CHR and ANT.

## Supplemental Materials

Supplementary data for this paper are available on-line only at http://jmb.or.kr.

## Figures and Tables

**Table 1:** Strains and plasmids used in this study.

Strain or plasmid	Characteristics	Sources or reference
*Corynebacterium glutamicum* ATCC13032		
Inha304	Δ*aroK*Δ*qsuB*Δ*pykA1*Δ*qsuD*	[19]
Inha310	Δ*aroK*Δ*qsuB*Δ*pykA1*Δ*qsuD*/pECBFG	[19]
Inha340	Inha304Δ*aroK*::*aroK*Δ*trpE*&*trpG*	This study
Inha341	Inha304Δ*aroK*::*aroK*Δ*trpE*&*trpG*&*trpD*ΔNCgl0819Δ*pabAB*Δ*entC*	This study
Inha342	Inha353Δ*trpE*&*trpG*	This study
Inha343	Inha354Δ*trpE*&*trpG*	This study
Inha344	Inha343Δ*iolR*::*Ptuf_ppgK*&*glk*	This study
Inha345	Inha344Δ*ack*&*pta*	This study
Inha350	Inha304Δ*aroK*::*aroK*Δ*trpD*	This study
Inha351	Inha350ΔNCgl0819Δ*pabAB*Δ*entC*	This study
Inha352	Inha351Δ*aroF*::*Psod_*E*aroG*&*EaroH*	This study
Inha353	Inha352Δ*aroG*::*Psod_aroF*&*aroB*	This study
Inha354	Inha353Δ*aroE*::*Psod_EaroE*&*qsuC*	This study
Inha355	Inha354Δ*iolR*::*Ptuf_ppgK*&*glk*	This study
Inha356	Inha355Δ*ack*&*pta*	This study
Plasmid		
pJYS3	pBL1^ts^ *oriV_C.glu_*. Kn^r^ pSC101 *oriV_E.coli_* PlacM_FnCpf1	This study (Addgene: 85542)
pJYS3_Δ*aroK*::*K*	pJYS3 containing Pj23119-crRNA targeting Δ*aroK*, 1 kb upstream and downstream homologous arms of *aroK* gene, *aroK* gene	This study
pJYS3_Δ*trpEG*	pJYS3 containing Pj23119-crRNA targeting *trpE*, 1 kb upstream and downstream homologous arms of *trpE* and *trpG* genes	This study
pJYS3_Δ*trpD*	pJYS3 containing Pj23119-crRNA targeting *trpD*, 1 kb upstream and downstream homologous arms of *trpD* gene	This study
pJYS3_ΔNCgl0819	pJYS3 containing Pj23119-crRNA targeting NCgl0819, 1 kb upstream and downstream homologous arms of NCgl0819 gene	This study
pJYS3_Δ*pabAB*	pJYS3 containing Pj23119-crRNA targeting *pabAB*, 1 kb upstream and downstream homologous arms of *pabAB* gene	This study
pJYS3_Δ*entC*	pJYS3 containing Pj23119-crRNA targeting *entC*, 1 kb upstream and downstream homologous arms of *entC* gene	This study
pJYS3_Δ*aroF*::*Psod_EaroG*&*EaroH*	pJYS3 containing Pj23119-crRNA targeting *aroF*, 1 kb upstream and downstream homologous arms of *aroF* gene, *sod* promoter, *aroG* and *aroH* gene from *E. coli*	This study
pJYS3_Δ*aroG*::*Psod_aroF*&*aroB*	pJYS3 containing Pj23119-crRNA targeting *aroG*, 1 kb upstream and downstream homologous arms of *aroG* gene, *sod* promoter, *aroF* and *aroB* gene from *C. glutamicum*	This study
pJYS3_Δ*aroE*::*Psod_EaroE*&*qsuC*	pJYS3 containing Pj23119-crRNA targeting *aroE*, 1 kb upstream and downstream homologous arms of *aroE* gene, *sod* promoter, *aroE* gene from *E. coli* and *qsuC* gene from *C. glutamicum*	This study
pJYS3_Δ*iolR*::*Ptuf_ppgK*&*glk*	pJYS3 containing Pj23119-crRNA targeting *iolR*, 1 kb upstream and downstream homologous arms of *iolR* gene, *tuf* promoter, *ppgK* and *glk* gene from *C. glutamicum*	This study
pJYS3_Δ*ack*&*pta*	pJYS3 containing Pj23119-crRNA targeting ack, 1 kb upstream and downstream homologous arms of ack and pta genes	This study
