# Supplementary material for: CRISPR-Driven Genome Engineering for Chorismate- and Anthranilate-Accumulating Corynebacterium Cell Factories
Source: J Microbiol Biotechnol. 2023 Jul 17;33(10):1370–5. doi: 10.4014/jmb.2305.05031 (PMC10619553; doi:10.4014/jmb.2305.05031)
Supplement: Supplementary file 1 [file jmb-33-10-1370-supple.pdf]

J. Microbiol. Biotechnol.
https://doi.org/10.4014/jmb.2305.05031

CRISPR-driven Genome Engineering for Chorismate- and Anthranilate-Accumulating *Corynebacterium* Cell Factories

Hye-Jin Kim, Si-Sun Choi, and Eung-Soo Kim*

Department of Biological Sciences and Bioengineering, Inha University, Incheon 22212, Korea

Supplementary Figures and Table

Figure 1. Genome re-design to produce CHR and ANT in *C. glutamicum*

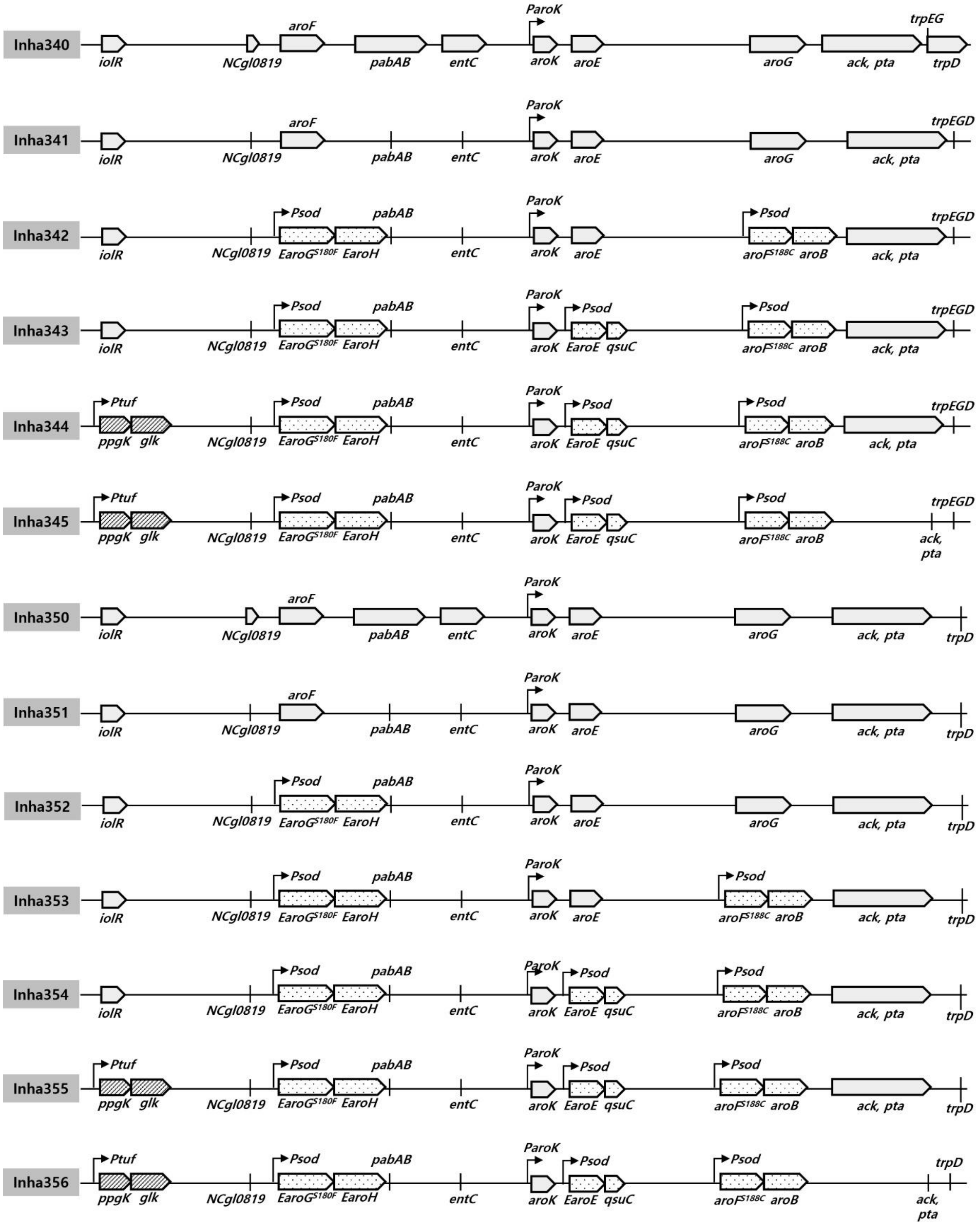

Supplementary Figure 2. Complementation of the shikimate kinase for production of chorismate and anthranilate based on shikimate overproducing strain (Inha310) (A) Scheme of shikimate kinase (encoding *aroK* gene) complementation, (B) Colony PCR results to verify complementation of target gene.

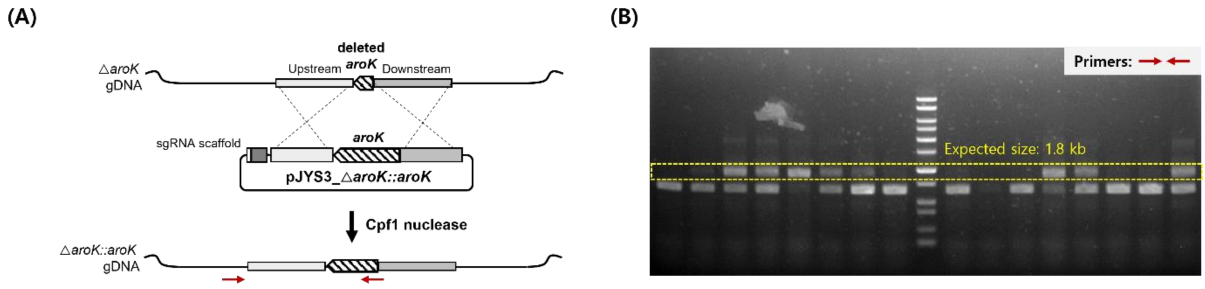

Supplementary Figure 3. Development of chorismate and anthranilate producing strain through deletion of anthranilate synthases (TrpE (NCgl2927) and TrpG (NCgl2928)) and anthranilate phosphoribosyltransferase (TrpD (NCgl2929)). (A) Scheme for deletion of *trpE* and *trpG* genes, (B) Scheme for deletion of *trpD* gene, (C) PCR confirmation to verify target gene deletion.

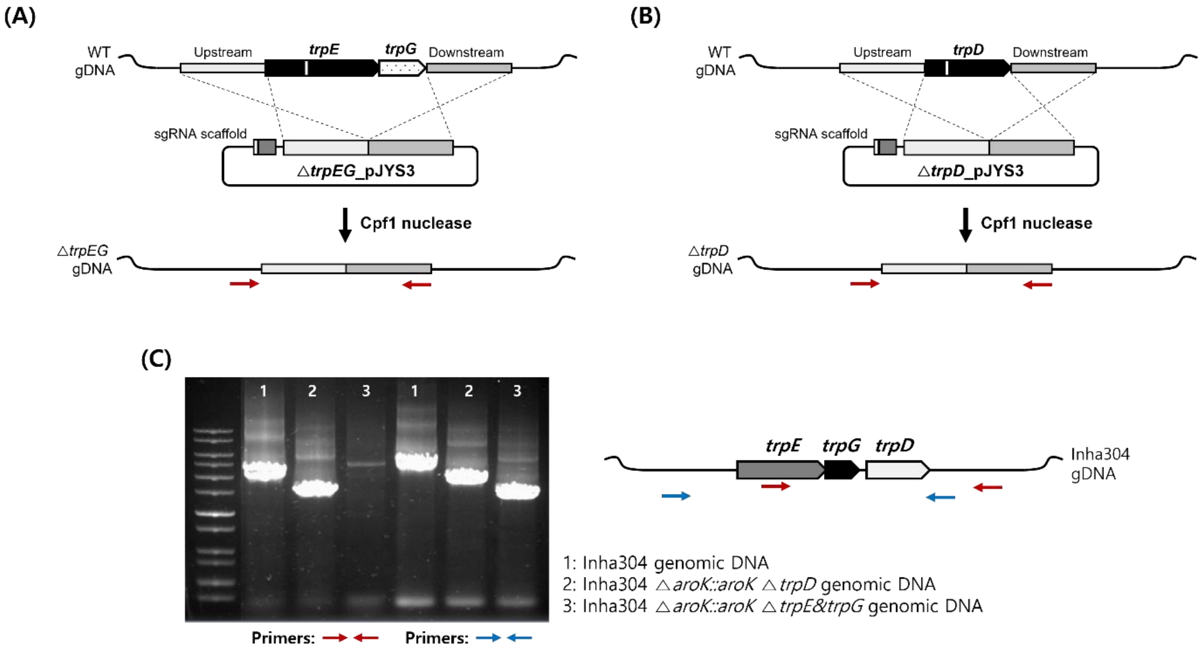

Supplementary Figure 4. Deletion of chorismate metabolic genes in anthranilate producing strain (Inha350). (A) Scheme for deletion of NCgl0819 gene (left) and PCR confirmation to verify NCgl0819 gene deletion (right), (B) Scheme for deletion of NCgl0955 gene (left) and PCR confirmation to verify NCgl0955 gene deletion (right), (C) Scheme for deletion of NCgl1243 gene (left) and PCR confirmation to verify NCgl1243 gene deletion (right).

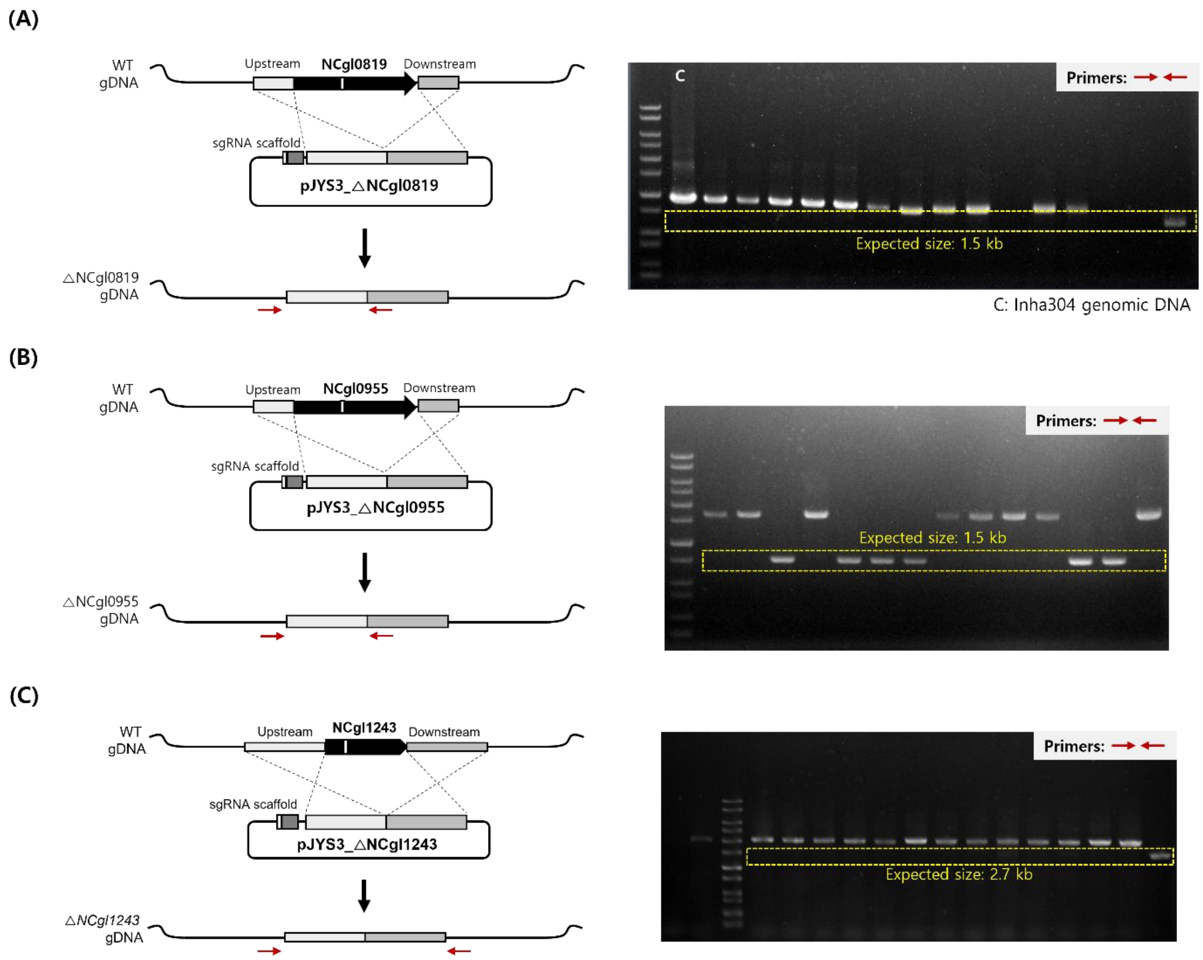

Supplementary Figure 5. Reconstruction of the shikimate pathway genes. (A) Scheme for substitution of feedback inhibition resistant *aroG* gene and *aroH* gene from *E. coli* for *aroF* gene in *C. glutamicum* (top), PCR confirmation to verify *aroF* gene replacement (bottom), (B) Scheme for substitution of feedback inhibition resistant *aroF* gene and *aroB* gene from *C. glutamicum* for *aroG* gene in *C. glutamicum* (top), PCR confirmation to verify *aroG* gene replacement (bottom), (C) Scheme for substitution of *aroE gene* from *E. coli* and *qsuC* gene for *aroE* gene in *C. glutamicum* (top), PCR confirmation to verify *aroE* gene replacement (bottom).

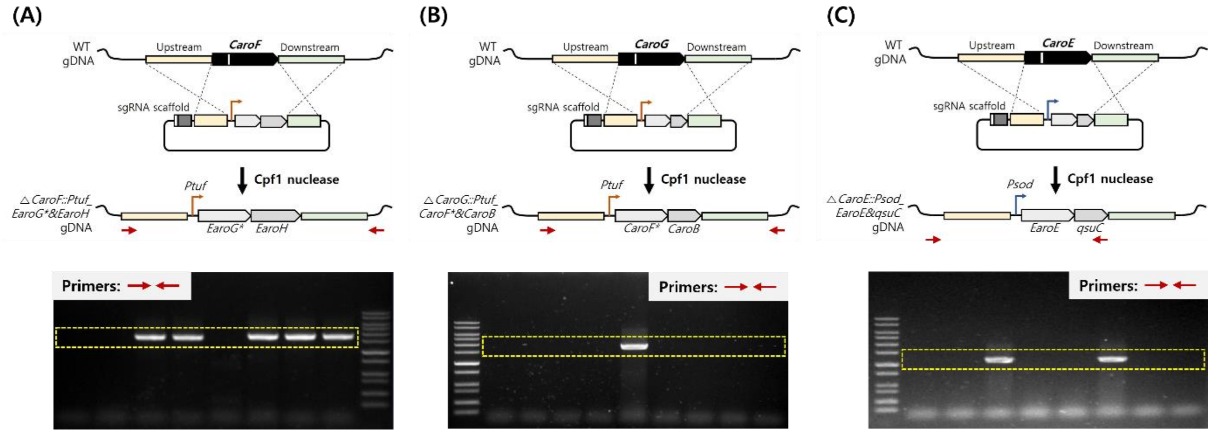

Supplementary Figure 6. Optimization of the central carbon metabolic genes. (A) Scheme for substitution of *ppgK* gene and *glk* gene for *iolR* gene (top), PCR confirmation to verify *iolR* gene replacement (bottom), (A) Scheme for deletion of *ack* gene and *pta* gene (top), PCR confirmation to verify *ack and pta* genes deletion (bottom).

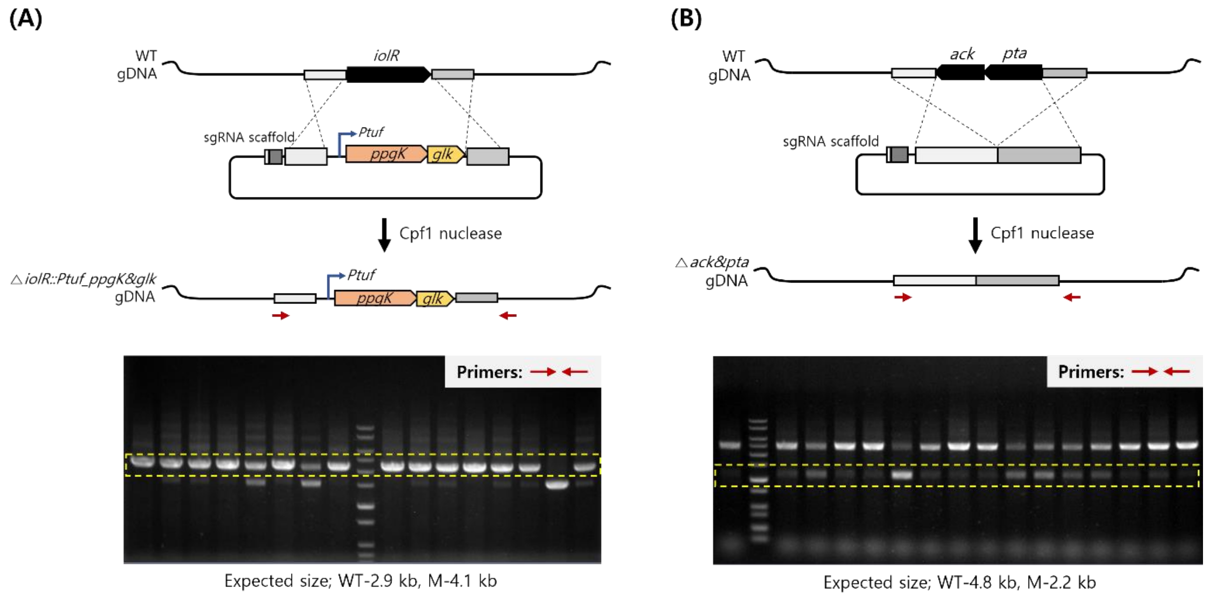

Supplementary Table 1. Primer pairs for the construction of plasmids and target gene amplification used in this study.

Purpose		Forward(F) / Reverse(R)
aroK complementation	sgRNA scaffold	5’-actgagcctttcgttttatttgcaacactttacgctccatcctctatctacaacagtagaaattcgg-3’
		5’-tctagattgacagctagctca-3’
	Upstream & aroK & Down stream	5’-tgagctagctgtcaatctagaatggactacagcaggtgaat-3’
		5’-actgttcaccgggcctctagagattgcctcataagcactctg-3’
	Complement CK	5’-aatttctactgtgcctcgcg-3’
trpEG deletion	sgRNA scaffold	5’-actgagcctttcgttttatttggggagcgtttcaaaggtttctaaatctacaacagtagaaattcgg-3’
		5’-tctagattgacagctagctca-3’
	Upstream	5’-tgagctagctgtcaatctagatactcgctggaatattggtgg-3’
		5’-cttagcactgactcaaacttgcgcagcacttcgat-3’
	Downstream	5’-tgagtcagtgctaagcccaa-3’
		5’-actgttcaccgggcctctagacgacgatgctttccaacact-3’
trpD deletion	sgRNA scaffold	5’-cctttcgttttatttgccaccagtaccagcggaatctagatctacaacagtagaaattcgga-3’
		5’-tctagattgacagctagctca-3’
	Upstream	5’-tgagctagctgtcaatctagactcaagttcaccgctgctaa-3’
		5’-attattactagtcatgaatcaaatcctttttttattagttcg-3’
	Downstream	5’-atgactagtaataatctgcccaca-3’
		5’-actgttcaccgggcctctagatagcccagtcgaatacttcc-3’
	Deletion CK	5’-aattcaccgaagtatgcaggc-3’
NCgl0819 deletion	sgRNA scaffold	5’- actgagcctttcgttttatttgaattttgctgcgcatgggacgcgatctacaacagtagaaattcgg -3’
		5’-tctagattgacagctagctca-3’
	Upstream	5’-tgagctagctgtcaatctagataagcgatgatgtcacggattt-3’
		5’-acgtggcagaatagtgtgc-3’
	Downstream	5’-ggcagaatagtgtgcgttatccacaggtagaaaaatattta-3’
		5’-actgttcaccgggcctctagagatcgagaaggtaggtgact -3’
	Deletion CK	5’-tacctgtggataacgcacact -3’
NCgl0955 deletion	sgRNA scaffold	5’- actgagcctttcgttttatttcttggatgttttaactgtggtgggatctacaacagtagaaattcgg -3’
		5’-tctagattgacagctagctca-3’
	Upstream	5’-tgagctagctgtcaatctagaagccactgaacgagcttgat-3’
		5’-aaaacaacgagaaaagcaccgc-3’
	Downstream	5’-ttttctcgttgttttatgacgtacctcgtgtggg-3’
		5’-actgttcaccgggcctctagactatgaagccacagaccgc-3’
	Deletion CK	5’-actgtatgagggcttggaag-3’
NCgl1243 deletion	sgRNA scaffold	5’-actgagcctttcgttttatttcgtcaatgcagccatcaataccatatctacaacagtagaaattcgg-3’
		5’-tctagattgacagctagctca-3’
	Upstream	5’-tgagctagctgtcaatctagaagccttctcattgaacaccag-3’
		5’-ctgggattagaatggctaacc-3’
	Downstream	5’-ttagaatggctaacctcgcttggtttgtgagatgtg-3’
		5’-actgttcaccgggcctctagattatcgagttcatcaccgcct-3’
	Deletion CK	5’-aatggctaacctcgcttggtt-3’
AroF sutstitution	sgRNA scaffold	5’-cctttcgttttatttgatgtttccgtcagttccgttcttatctacaacagtagaaattcgga-3’
		5’-tctagattgacagctagctca-3’
	Upstream	5’-agctgtcaatctagaggaaattcttggcactgagc-3’
		5’-cgatttcaagatatctcgtgctgcgaacatagtttg-3’
	Tuf promoter	5’-gatatcttgaaatcggctttcaac-3’
		5’-tgtatgtcctcctggacttc-3’

	EaroG mutation	5’-ccaggaggacatacaatgaattatcagaacgacgatttacg-3’
		5’-gtcagcgagatattgtagggtgatcatatcgagaaac-3’
		5’-acaatatctcgctgacctga-3’
		5’-gtcagttctgttcattacccgcgacgcgctttta-3’
	EaroH	5’-atgaacagaactgacgaactcc-3’
		5’-acagtctagcgcccttcagaagcgggtatctaccg-3’
	Downstream	5’-agggcgctagactgttaaatg-3’
		5’-actgttcaccgggcctctagaagagatcggtatcggtaatgaat-3’
	Substitution CK	5’-gctatggataagccaggaatc-3’
AroG substitution	sgRNA scaffold	5’-cctttcgttttattttgtgagttggacagttgatatcccatctacaacagtagaaattcgga-3’
		5’-tctagattgacagctagctca-3’
	Upstream	5’-tagctgtcaatctagaggtactttgatttcatcacagttg-3’
		5’-cgatttcaagatatccccacacctttatctatttggat-3’
	Tuf promoter	5’-gatatcttgaaatcggctttcaac-3’
		5’-tgtatgtcctcctggacttc-3’
	CaroF mutation	5’-ccaggaggacatacaatgagttctccagtctcactc-3’
		5’-gaaaccaattggcatgcacatcccagaagccagctgg-3’
		5’-atgccaattggtttcaagaa-3’
		5’-attacttggctgctgctcggc-3’
	CaroB	5’-gcagcagccaagtaatgagcgcagtgcagattttc-3’
		5’-ttagtggctgattgcctcataag-3’
	Downstream	5’-gcaatcagccactaattctcccaacctatggaatgg-3’
		5’-actgttcaccgggcctctagagctacactgcttgcaaggat-3’
	Substitution CK	5’-attggtgccatcctgtttgc-3’
AroE substitution	sgRNA scaffold	5’-cctttcgttttatttagaagtcacacgagccaaaggcctatctacaacagtagaaattcgga-3’
		5’-tctagattgacagctagctca-3’
	Upstream	5’-tgagctagctgtcaatctagatgaccaacgttttcgcagac-3’
		5’-cacctcctcaacaaaactcct-3’
	Sod promoter	5’-tttgttgaggaggtgaattgaggaagccttatgccc-3’
		5’-aaaaacagcataggtttccatgggtaaaaaatcctttcgtaggt-3’
	EaroE	5’-atggaaacctatgctgtttttgg-3’
		5’-tcacgcggacaattcctcc-3’
	qsuC	5’-ggaattgtccgcgtgatgcctggaaaaattctcctcc-3’
		5’-ctactttttgagatttgccagga-3’
	Downstream	5’-aatctcaaaaagtagttattcgctgatccttatcggtt-3’
		5’-actgttcaccgggcctctagatgacgatcgcttcactcatc-3’
iolR substitution	sgRNA scaffold	5’-cctttcgttttatttcgcgttgcattggataattccggcatctacaacagtagaaattcgga-3’
		5’-tctagattgacagctagctca-3’
	Upstream	5’-tgagctagctgtcaatctagaatgagtttcaccggagcgta-3’
		5’-tgagctagctgtcaatctagaatgagtttcaccggagcgta-3’
	Tuf promoter	5’-gatatcttgaaatcggctttcaac-3’
		5’-tgtatgtcctcctggacttc-3’
	ppgK	5’-ccaggaggacatacaatgactgagactggatttggaat-3’
		5’-ttatggggtgaggtgttggtt-3’
	glk	5’-cacctcaccccataatgccacaaaaaccggccag-3’
		5’-atttacatgagcttgctagttggcttccactacaga-3’
	Downstream	5’-caagctcatgtaaatgtgttagg-3’
		5’-actgttcaccgggcctctagacgcattcttccttgttgccaa-3’
	Substitution CK	5’-acaccgcgttccaacagtg-3’
ack & pta deletion	sgRNA scaffold	5’-cctttcgttttatttgttaatgatttccgtgaactgcggatctacaacagtagaaattcgga-3’
		5’-tctagattgacagctagctca-3’
	Upstream	5’-tgagctagctgtcaatctagacaaagatttcgaagacctcga-3’
		5’-ttaggatccaccacaaatcgc-3’
		5’-tgtggtggatcctaagatgacacttcaggcttgtg-3’

	Downstream	5’-actgttcaccgggcctctagaccttagcctgattcttagcca-3’
	Deletion CK	5’-gaatggtcaagttccttcagc-3’
